# Notes and News

**Published:** 1888-07-07

**Authors:** 


### NOTES AND NEWS.

Collected and Communicated.

Their Royal Highnesses the Prince and Princess of Wales have fixed Tuesday, the 17th inst., as the date for the postponed ceremony of opening the new buildings of the Great Northern Central Hospital.

James Cagney, M.D., M.A., has been appointed assistant physician and registrar to the Hospital for Epilepsy and Paralysis, and other Diseases of the Nervous System, 32, Portland Terrace, Regent's Park.

Mr. James Mason, of Whitney, Oxon, has become a life member of The Hospitals' Association by the payment in one sum of £21. Mrs. Charles Myers, of Bishop's Waltham, has also contributed £21 as a life member.

The *St. Stephen's Review* silver-wedding offering on behalf of the Prince and Princess of Wales to the Chelsea Hospital for Women amounts to the sum of £184 19*s*., which has been handed to the hospital authorities.

A public meeting is to be held at the Mansion House on July 6th in support of the Building Fund of the new Hospital for Women in Marylebone Road. The physicians for in and for out patients are ladies, and it is hoped that £20,000 will be collected.

A Children's Convalescent Home is in contemplation at Eastbourne. It is proposed to erect the new home as a memorial to Miss Harriet Brownlow Byron, who founded the magnificent convalescent hospital already existing there, with which the new institution will be in connexion. An urgent appeal is now being made for funds.

It is proposed to make some additions to the Herbert Convalescent Home, Bournemouth, which will restore to the use for which it was originally destined, a ward which has of late years served as a chapel. A new chapel will be built, as well as two additional day-rooms for patients. Lord Henry Somerset has presented to the committee the chairs, tables, fittings, etc., and American organ from his private convalescent home at Bournemouth.

Our own special schoolboy correspondent sends the following account of the Thanet election: The Conservative candidate for Thanet died, so we had an election. I wore the Conservative colours—blue and yellow—because I knew that father was a great Conservative. The Conservative candidate got in; he beat by a lot of votes. We were going for a walk yesterday afternoon, and we met Lowther, the Conservative. He said, "Cheer, boys, cheer," so we cheered him loudly. And again we saw a Conservative, and cheered him very loudly. I am sure that father will be pleased to hear about the Conservative.

Miss Traill Christie, of 6, Russell Road, Kensington, has sent to the editor of *Toilers of the Deep*, the monthly journal of the Mission to Deep Sea Fishermen, a first instalment of £20 towards the fund for the floating hospital, which it is desired to found for the fishermen of the North Sea.

We understand that a provisional committee has been appointed by the Queen for the purpose of organising, in conjunction with the Duke of Westminster, Sir Rutherford Alcock, and Sir James Paget, a scheme for establishing with the Women's Jubilee Fund a system of nursing the sick poor in connexion with St. Katherine's Hospital, and that the committee held its first meeting on the 26th ult.

A Ladies' Committee has been formed, under the auspices of the Mayor and Mayoress of Newcastle-on-Tyne, to assist in carrying on the work of the Royal Infirmary. At a meeting called by the Mayor, the Mayoress presided, and it was resolved that a committee consisting exclusively of ladies be formed forthwith. The special duties of the members of the committee will be to visit ths hospital every week in turn, to assist in the raising of a fund for the support of the hospital, and to collect and distribute clothing of all kinds among the poorer patients.

In raising money for charitable or any other purposes, the cost of collection is always one of the most important circumstances for consideration. Some of the methods employed by hospitals are scandalously wasteful. There have been known bazaars in which a third of the total sum raised has been thrown away in expenses. The Birmingham Saturday Fund in this respect, as well as in the noble amount collected, has set an excellent example to other similar organisations. The total expenses incurred in the collection of nearly £8,000 were less than £400, or a fraction over 5 per cent.

Drawings for an addition to Ancoats Hospital, Manchester, have been prepared by Messrs. Pennington and Bridgen, architects. It is intended to adapt the present structure in Mill Street to the Out-patients' Dispensary, with the necessary consulting and dressing rooms, and to administrative purposes, providing all the requisite accommodation for the resident medical staff, nurses, and servants, and retaining only a ward for 12 children on the first floor. The remainder of the in-patients will be provided for in a pavilion of three storeys in the rear of the administrative block, the ground floor being used as day rooms, and the two upper floors as wards for 60 beds, thus making a total of 72 beds. The pavilion is connected with the administration by a wide corridor, and it is intended to build a new kitchen with its necessary adjuncts, together with an operating-room, laundry, and mortuary. All the corridors and staircases will be fireproof, and the hospital will be fully equipped with all the most recent constructive appliances.

A new hospital has been formed, an Animals' Institute, which has its local habitation in Wilton Place, S.W. The objects of the institute are to carry out the humane treatment of domesticated animals, the study of comparative pathology without vivisection, and the alleviation of pain and suffering in the lower animals. Gratuitous advice for animals will be given daily between the hours of nine a.m. and twelve noon; in-patients will be received in urgent and severe cases, and operations will be conducted under chloroform. There will also be an anæsthetic chamber for the painless destruction of aged and incurably diseased animals. Subscribers will receive tickets for distribution amongst the poor, whose animals will have treatment and medicine gratis on the owners producing their ticket at the institute. Among the list of patrons and subscribers we note the name of that well-known lover of animals, Miss Power Cobbe. We hope the Animals' Institute will do good service to our "poor relations" of the dumb creation.

THE following vacancies are announced this week, the amount of salary in each case being given after the name of the institution :—

**Resident Medical Officers.**

City of London Hospital for Diseases of the Chest, Victoria Park, E. Resident Clinical Assistant.
Derbyshire General Infirmary. Resident Assistant House Surgeon.—Board, and bonus of £10.
East Suffolk and Ipswich Hospital. House Surgeon, doubly qualified.—£80 per annum, with board.
General Infirmary, Northampton. Assistant House Surgeon. Candidates must be M.R.C.S.,E., and L.R.C.P., or L S.A., Lond.—£80 per annum, with board, etc.
Glasgow Hospital for Sick Children. Assistant House Surgeon.
Hospital for Women, Soho Square, W.C. House Physician, doubly qualified.—£75 per annum, with board, etc.
Hoxton House Asylum, London. *Locum tenens* to act as Assistant Medical Officer for July and August.—Two guineas a week, with board.
Kent County Asylum, Barming Heath. Third Assistant Medical Officer.—£120 per annum, with furnished apartments, &c.
North Staffordshire Infirmary. Assistant House Surgeon.—Board, etc.; no salary.
West Sussex, East Hants, and Chichester Infirmary. House Surgeon.—£100 per annum, with board, &c.
Worcester General Infirmary. House Surgeon, doubly qualified.—£100 per annum, with board, etc.

**Non-resident Medical Officers.**

Bristol General Hospital. Assistant Surgeon. Candidates must be Fellow or Member of the Royal College of Surgeons of England, or Graduate of in Surgery of a University of Great Britain or Ireland; and if he be not a Fellow of the Royal College of Surgeons of England, he shall give an undertaking in writing that he will become a Fellow of the said college within twelve months of his election, and that if he fail to obtain the said diploma within the stipulated period he will forthwith resign his appointment.
City of London Hospital for Diseases of the Chest, Victoria Park, E.—Assistant Physician. Candidates must be F. or M.R.C.P., Lond.
City of London Hospital for Diseases of the Chest, Victoria Park, E. Pathologist. Candidates must be registered, and not engaged in general practice.—£105 per annum.
Killybegs and Tribane. Admiralty Surgeon and Agent for these Coastguards Stations —£50 per annum.
Middlesex Hospital, W. Physician for Skin Diseases. Candidates must be F. or M.R.C.P.,Lond., or F.R.C.S., E.
Parish of Edrachillis, Sutherland.—£150 per annum, with free house.
Parish of Kirkmaiden. Medical Officer.—£65 per annum.
Plymouth Public Dispensary. Physician's Assistant.—£60 per annum. Candidates must be doubly qualified, and must not hold a poor law appointment.
St. George's-in-the-East Parish, London, Infirmary Workhouse. Medical Officer.—£300 per annum, and unfurnished house. Candidates must be doubly qualified.
University College Hospital. Surgical Registrar.

BIRMINGHAM seems to have established its claim to priority in the origination of the Hospital Sunday Fund, and now it appears to be determined to establish a claim to pre-eminence in the success of its Hospital Saturday operations. The grand total of £7,754 17*s.* 10*d.* is the result of last year's efforts. When it is remembered that London, with a population eight times that of Birmingham, raises less than £12,000 by its Hospital Saturday organisation, the conspicuous distinction of Birmingham is clearly seen. Either London is miserably deficient in liberality, or its provincial rival is the most generous of towns.

---

WITH a view to the construction of a simple and comprehensive scheme for the general application of the Pay System, or for the abandonment of the idea if further discussion shall seem to render it necessary, the Special Committee appointed by The Hospitals Association to deal with the subject earnestly solicit information and suggestions from responsible hospital managers both in this and other countries. The magnitude of the question and its importance, both to patients, hospital supporters, and the medical profession can hardly be over-estimated. If the subject be handled with intelligence, courage, and resolution, results of the most far-reaching and beneficial character will undoubtedly ensue.

THE Dental Hospital of London, Leicester Square, has received notice that a legacy of £2,000 has been bequeathed to it by the late Mr. Capel Carter.

---

MUCH interest has been aroused throughout the country by the recent discussion at The Hospitals Association of the Pay System as a new factor in hospital finance. The Council of the Association have reason to be gratified by the publicity given to their movement by the leading daily and weekly newspapers, both in London and the country, and by the many practical suggestions made in leaders which have been written on the subject. There is no doubt whatever that so far as the laity are concerned some form of the Pay System is decidedly favoured. The Hospitals Association has now before it a great opportunity, and the country looks to it with hopeful expectation to propose such a scheme as shall commend itself to the sober and reflecting among hospital supporters. It is to be deplored that whilst in the country generally, and nowhere more than in London, the parliamentary constituencies are fully alive to the importance of the questions at issue, the metropolitan Members, with the honourable exception of Lord Randolph Churchill and one or two others, seem to be entirely indifferent about the straits into which our noble medical charities have fallen.

---

THE Right Hon. Hugh C. E. Childers, M.P., presided at a large public meeting at the Bow and B.omley Institute on Tuesday, June 26th, on behalf of the London Hospital. The chairman was supported by the Rev. R. C. Billing, M.A. (rector of Spitalfields, and Bishop-Designate of Bedford), Captain Sir John Colomb, K.C.M.G., M.P., Samuel Montagu, Esq., M.P., J. Wootton Isaacson, Esq., M.P., Sir Edmund Hay Currie, E. Brigham, Esq., N.L.R., J. H. Buxton, Esq. (treasurer of the hospital), and F. C. Carr-Gomm, Esq. (chairman of the hospital). The question considered at the meeting was the work done by the hospital amongst the crowded population of the East-end, and with a view to inducing all residents and working men to assist the management in their endeavours to maintain the present efficiency of the hospital by clubs and subscriptions amongst themselves. The first resolution set forth that the London Hospital is carrying on a work of national importance in the East-end of London, and is worthy of the support of all classes. The second affirmed that the residents and employés of the East-end should endeavour—through their clubs and benefit societies, and by organised subscriptions amongst themselves—to aid the authorities by every means in their power to maintain the efficiency of the hospital. Special appeals for donations and subscriptions are only made by the hospital once every five years. At least £34,000 from voluntary contributions are required every year, and the committee earnestly appeal to the public for the same. The London Hospital is absolutely indispensable to the working population of East London. It is the largest general hospital in England. It is the largest children's hospital in London. It is the largest hospital for Jews in the metropolis. It is a special hospital for cancer, fistula, skin disease, diseases of the eye, ear, and throat, and obstetric maladies. The number of beds is 776. Over 104,000 patients were treated last year. The assured income is only £16,000, the necessary expenditure is £51,000, leaving an annual deficit of £35,000. The hospital is quite unsectarian; there is no religious sisterhood, and persons of all denominations are admitted either as patients or employés, perfect freedom being allowed to all, in religious matters. To carry on this great work there are twenty-five physicians and surgeons, five house physicians, five house surgeons, one resident accoucheur, as well as a number of students who assist in the work, and over two hundred nurses and sisters; there is a committee of management, a chaplain, an assistant chaplain, a house governor, a secretary, a steward, a housekeeper, a surveyor, &c.

